# Applications of Artificial Intelligence in the Control of Infectious Diseases in the Post-COVID Era: Scoping Review

**DOI:** 10.2196/84242

**Published:** 2025-11-17

**Authors:** Chanhee Kim, Robin Austin, Rebecca Wurtz, Connie White Delaney, Sripriya Rajamani

**Affiliations:** 1 School of Nursing, University of Minnesota, 308 Harvard St SE, Minneapolis, MN, 55455, United States, 1 651-278-7426; 2 School of Public Health, University of Minnesota, Minneapolis, MN, United States

**Keywords:** artificial intelligence, infectious disease control, public health practice, machine learning, large language models, health communication, nursing representation, implementation science

## Abstract

**Background:**

The COVID-19 pandemic exposed systemic vulnerabilities in public health infrastructure, underscoring the urgency for innovation in disease surveillance and emergency response. Artificial intelligence (AI) has emerged as a promising tool to enhance the accuracy, efficiency, and scalability of public health interventions. Yet, there remains a limited understanding of how AI has been applied in real-world infectious disease control and who is contributing to its development and implementation.

**Objective:**

This scoping review aimed to map current applications of AI in public health practice for infectious disease control since 2020. Specifically, it examined (1) the types of AI tools in use, (2) their purposes and implementation contexts, and (3) the professional and institutional actors leading these efforts, including the role of nurses.

**Methods:**

Using the Joanna Briggs Institute’s population, concept, and context framework, a structured search in Ovid MEDLINE was conducted, which was guided by the “5Cs” framework for health emergency preparedness from the World Health Organization (WHO). The search focused on English-language, peer-reviewed studies from 2020 that used AI tools for infectious disease control within real-world public health practice. Nonoriginal articles, simulation-only studies, and studies that lacked real-world implementation were excluded.

**Results:**

Out of 600 screened studies in Ovid MEDLINE, 10 met the inclusion criteria. Two major AI types were identified: machine learning (ML) algorithms and language-based tools such as chatbots and large language models. ML tools supported outbreak detection, risk stratification, and resource allocation, while language-based tools promoted health communication, particularly around immunization and HIV prevention. Studies were conducted in a diverse range of countries, including several low- and middle-income countries, and used national datasets or surveillance systems. Despite nurses comprising half of the global health workforce, no nursing-affiliated authors were found among first or corresponding authors, and no nurses were represented in the broader authorship of the included studies.

**Conclusions:**

AI technologies are being increasingly applied to support public health responses to infectious diseases, with applications ranging from predictive analytics to real-time public engagement. However, adoption remains limited in scale, scope, and professional diversity. The near-total absence of nursing participation in AI-related public health research is particularly striking and represents a missed opportunity for inclusive innovation. Strengthening implementation research and advancing informatics education among nursing professionals are critical next steps to ensure that AI tools reflect the realities of public health practice and promote equitable outcomes.

## Introduction

According to the World Health Organization (WHO), approximately 7.1 million deaths due to COVID-19 have been reported as of July 2025 [1]. This pandemic exposed the vulnerabilities and systemic shortcomings of global public health infrastructure, underscoring the urgent need for structural reforms [2]. COVID-19 clearly highlighted the limited and uncoordinated pandemic preparedness, leaving health systems unprepared and overwhelmed during a rapidly spreading outbreak [3].

It is evident that public health, like other facets of modern life, needs comprehensive modernization to ensure resilience and effectiveness in the face of future global health crises [4]. The potential of artificial intelligence (AI) has emerged as a powerful engine of transformation. While there is currently no universally accepted definition of AI in the field of public health [5], the WHO has described it as “the performance by computer programs of tasks that are commonly associated with intelligent beings” [6]. The application of AI to public health has started to reshape how agencies and the practitioners of health manage disease surveillance and health services, as well as how they predict, prevent, and optimize health outcomes at the population level [7]. Predictive modeling, real-time analytics, natural language processing (NLP), and automated support systems for decisions are some areas in which AI has shown great potential for enhancing accuracy, efficiency, and scalability [7-9]. In clinical and medical research, AI has been applied across diverse areas of health care, including disease diagnostics, infectious disease forecasting, medical imaging interpretation, and drug discovery [7,10,11]. However, in public health, it is still unclear which AI tools are being used in practice and to what extent.

As AI continues to revolutionize how data are collected, interpreted, and used for the control of infectious diseases, understanding how AI augments the practice of public health becomes critical. Mapping the landscape of AI applications to the control of infectious diseases provides a clear picture of the use of these technologies. This understanding is also relevant to the frontline workers of the public health workforce and the interprofessional teams of nurses, data scientists, epidemiologists, and public health practitioners, who are responsible for developing and delivering AI-based interventions. Interprofessional collaboration promotes the development of innovation, closing the gap between research and the frontline application of solving real-world health challenges [12].

Given their frontline roles, nurses need to be an integral part of interprofessional teams in AI development and implementation in health care and public health. Despite their vital role in health care delivery systems, nurses are often underrepresented in public health [13]. Nurses comprise approximately 50% of the global health workforce and have historically not only contributed to clinical care but also supported entire public health systems [14]. Nonetheless, the limited focus on nurses in public health underscores the need to incorporate nursing leadership and nursing expertise as integral components of equitable and sustainable systems [13].

In response to these challenges, this review aims to explore how AI has been used to support the control of infectious diseases in public health and how it strengthens responses to such challenges, particularly by examining the contributors in this emerging field. Specifically, it outlines the current landscape of AI applications in three key areas: (1) identifying the types of AI tools currently used for infectious disease control; (2) examining the specific infectious disease strategies where these tools are applied; and (3) analyzing who is leading and contributing to these AI-driven efforts, across countries, institutions, and professional fields, including nursing.

## Methods

### Structuring the Review Using the Population, Concept, and Context Framework

As the review’s objective was to map the landscape of AI applications in infectious disease control, the population, concept, and context (PCC) framework recommended by the Joanna Briggs Institute (JBI) was adopted. The PCC framework is most suitable for reviews intended to clarify major concepts, identify areas of knowledge gaps, and summarize how research is conducted regarding a focus topic [15]. The review focuses on the actors involved in the control of infectious diseases (population), the use of AI tools for the control of infectious diseases (concept), and the postpandemic era of the practice of public health since the year 2020 (context).

Using the PCC framework, the following research questions were articulated:

1. What types of AI tools are currently being used to address infectious disease problems in public health?
2. How are these AI tools being applied across different stages and settings of infectious disease control?
3. Who is leading and participating in these AI-enabled efforts—specifically, to what extent are nursing professionals represented in the published literature?

### Key Concept Definitions

To achieve conceptual clarity and provide consistent analysis, this review defined its 3 central concepts: AI, infectious diseases, and public health interventions.

AI is defined as the use of computational techniques that enable machines to perform tasks that typically require human intelligence [16]. This includes, but is not limited to, machine learning (ML), deep learning, NLP, neural networks, and large language models (LLMs) [16,17]. As the WHO pointed out, AI is now an integral part of how health systems operate [6].

Infectious diseases are illnesses caused by pathogenic organisms or their toxic byproducts, transmitted to susceptible hosts through contact with infected individuals, animals, or contaminated surfaces or objects [18]. Due to the possibility of rapid spread among populations and across borders, infectious diseases pose a constant and high-priority threat to global health systems [19]. The urgency of timely detection, decision-making, and coordinated responses in managing infectious disease outbreaks has made this domain a natural candidate for the application of advanced technologies such as AI [20].

Public health interventions refer to coordinated strategies implemented to achieve targeted outcomes related to disease prevention and population health improvement [21]. Infectious diseases, more than others, frequently need swift, orderly responses, which can be targeted at either the infecting source (the isolation or elimination of infectious material) or vulnerable persons (through quarantine, risk communication, or immunization) [22,23].

### WHO’s 5Cs Framework for Health Emergency Preparedness and Response

For identifying studies that address all 3 core concepts (AI, infectious diseases, and public health interventions), a comprehensive search strategy was required. Although choosing keywords for AI and infectious diseases is relatively straightforward, the concept of public health interventions is more expansive and diverse. To address this complexity, the WHO’s “5Cs” framework for health emergency preparedness and response was adopted as a conceptual guide [24]. The framework organizes intervention efforts into 5 domains: Emergency Coordination, Community Protection, Collaborative Surveillance, Safe and Scalable Care, and Access to Countermeasures. These elements capture the practical requirements of real-world infectious disease response and, as such, represent a suitable resource for informing this review.

Each of the domains was then related to applicable infectious disease control concepts and subsequently linked to Medical Subject Headings (MeSH) (Table 1). For example, the domain of Collaborative Surveillance was linked to terms such as “Population Surveillance,” “Contact Tracing,” and “Early Diagnosis.” Some of the terms were applicable in more than one domain, like “Emergency Medical Services” and “Diagnostic Tests, Routine,” as they span multiple areas of public health response rather than being limited to a single function.

**Table 1.** Mapping of the domains of the World Health Organization’s 5Cs strategy to infectious disease concepts and MeSH^a^ terms.

5Cs strategy domains	Infectious disease concepts	Related MeSH terms
Emergency Coordination	Emergency preparedness Outbreak response Incident management	Disaster Planning Emergency Medical Services Disease Outbreaks Public Health Administration
Community Protection	Vaccination Risk communication Health education Community engagement	Vaccination Immunization Programs Health Education Health Communication Community Participation
Collaborative Surveillance	Surveillance Contact tracing Screening Early detection	Population Surveillance Contact Tracing Diagnostic Tests, Routine Mass Screening Early Diagnosis
Safe and Scalable Care	Infection control Health system capacity Workforce protection	Infection Control Health Services Delivery of Health Care Emergency Medical Services
Access to Countermeasures	Diagnostics Therapeutics Supply chains Resource allocation	Diagnostic Tests, Routine Pharmaceutical Services Pharmaceutical Preparations Drug Delivery Systems Resource Allocation

a MeSH: Medical Subject Headings.

### Search Strategy

To enhance the comprehensiveness of the search and minimize the risk of omitting relevant literature, the MeSH terms identified in relation to each WHO 5Cs domain were further examined in terms of their hierarchical structure. Wherever possible, broader parent terms were included in the search strategy to ensure that conceptually related subheadings were not excluded. For example, “Population Surveillance” and “Mass Screening” are nested under “Public Health Practice,” and in this case, “Public Health Practice” was included. The search strategy is presented in Multimedia Appendix 1.

**Figure 1.**
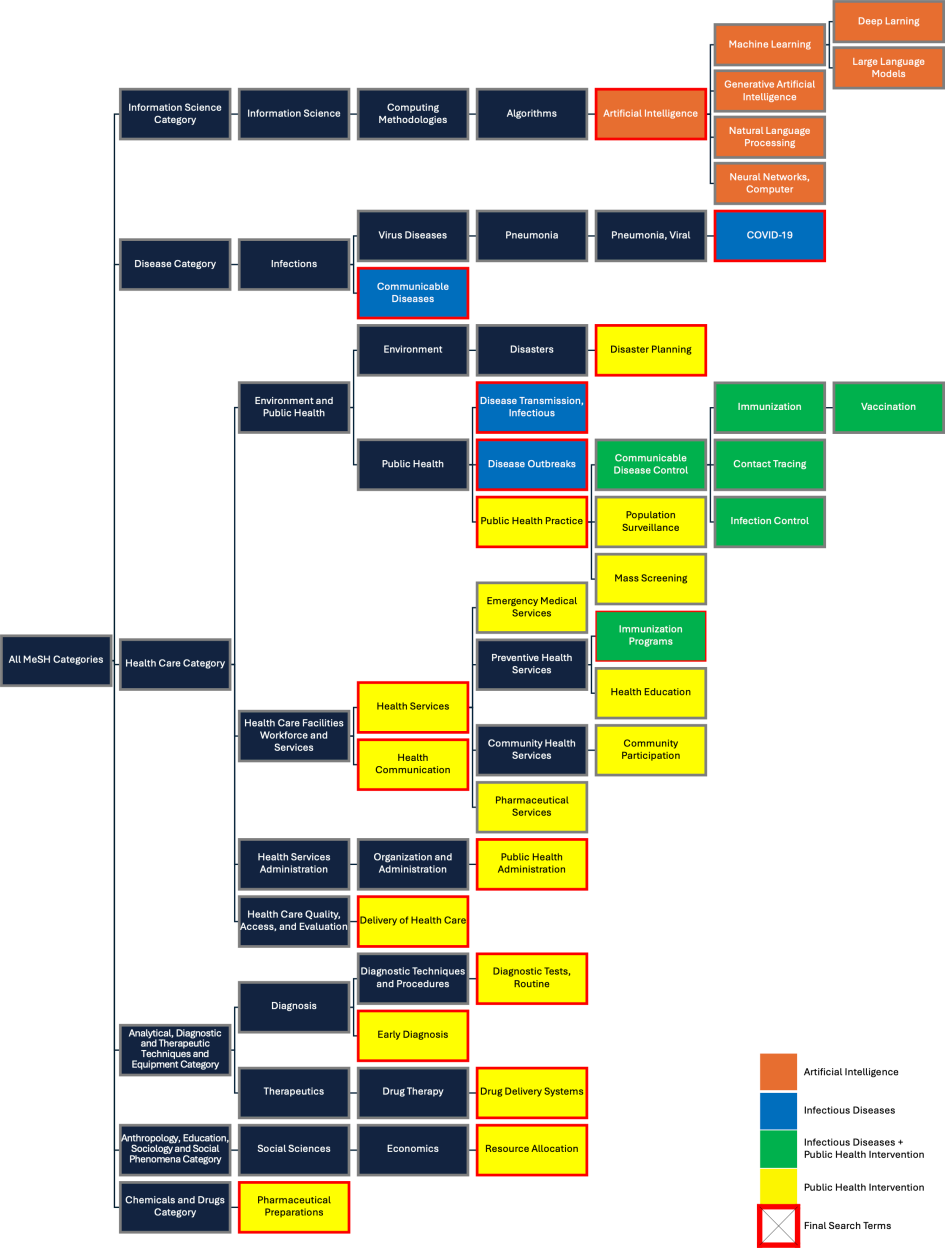
Hierarchical structure of reviewed and selected Medical Subject Headings (MeSH) for the final search strategy.

Figure 1 visualizes the final set of MeSH terms considered for inclusion, mapped onto their official hierarchical structure. Colors indicate conceptual categories: orange for AI terms, blue for infectious disease terms, yellow for public health intervention terms, and green for terms relevant to both infectious diseases and public health interventions. Terms in red boxes were ultimately selected for inclusion in the final search strategy.

These finalized MeSH terms were combined to construct a conceptually aligned search strategy in Ovid MEDLINE, to reflect the 3 core domains of this review—AI, infectious diseases, and public health interventions. The explosion function enabled the incorporation of studies that included only narrower terms in the search results. For example, since “Public Health Practice” was included in the search strategy with the explosion function enabled, studies with keywords, such as “Mass Screening” or “Population Surveillance*,*” which are nested under “Public Health Practice,” were also captured in the search results.

For the concept of AI, the search strategy included the MeSH term “Artificial Intelligence” with the explosion function enabled. To ensure that AI was a central focus of investigation, the search was limited to studies that explicitly mentioned AI-related terms in the article title, including “Artificial Intelligence,” “AI,” “Large Language Model*,” “Machine Learning,” “Deep Learning,” “Natural Language Processing,” “Neural Network*,” “Generative Artificial Intelligence,” “ChatGPT*,” and “Chatbot*.”

For the second core concept, infectious diseases, the search strategy incorporated MeSH terms such as “*Disease Transmission, Infectious,” “*Infectious Diseases,” “*Disease Outbreaks,” and “*COVID-19.”

Lastly, to identify relevant studies involving public health interventions, MeSH terms selected based on the WHO’s 5Cs framework were included: “*Disaster Planning,” “*Public Health Practice,” “*Health Services,” “*Health Communication,” “*Public Health Administration,” “*Delivery of Health Care,” “*Diagnostic Tests, Routine,” “*Early Diagnosis,” “*Pharmaceutical Preparations,” “*Drug Delivery Systems,” and “*Resource Allocation.”

However, some terms, such as “Communicable Disease Control” and “Immunization Programs,” inherently encompass the concepts of infectious diseases and public health interventions. To account for this overlap, search results for these combined terms were integrated with results from the separate concepts of infectious diseases and public health interventions using an OR operator, and the final set was combined with AI-related terms using an AND operator.

Finally, the search was limited to English-language, human-subject studies published from 2020 onward to reflect the post–COVID-19 landscape. Nonoriginal publications, such as case reports, commentaries, editorials, letters, meta-analyses, news articles, and review studies, were excluded from the search results.

### Inclusion and Exclusion Criteria

The PCC framework was subsequently reapplied to guide the screening process and determine study eligibility. For the *population* element, the first step in screening was to determine whether the studies were concerned with infectious diseases affecting humans. Studies focusing on nonhuman pathogens (eg, infectious waste disposal) or environmental contamination were excluded.

Next, in line with the *concept* element, an assessment was conducted to determine whether the tools used in public health interventions were genuinely based on AI. Studies that involved only basic digitalization or mechanization, without integrating AI functionalities, were not considered eligible.

The *context* of the review focused on public health practice after the pandemic. In applying this criterion, particular attention was paid to whether each study explicitly addressed a public health domain. For example, if the study covered the AI tools that were used to support clinical decision-making in emergency department triage or to optimize resource allocation within health care facilities, the study was excluded, as these applications fall outside the scope of public health practice as defined in this review.

Another key element related to the context was the practical implementation of AI. Studies were excluded if the AI tools were not applied to real populations, did not utilize official public health data, or lacked real-world implementation. This included studies limited to development or analysis only, such as simulation or predictive modeling. However, studies were deemed eligible if they had a clear practical application to public health. For example, studies that used authorized public health systems or infrastructure for data, such as national surveillance platforms or electronic health records, were included, as they demonstrated direct relevance to and integration within existing public health practice.

### Data Extraction

The initial screening was performed using Rayyan, a freely accessible web-based tool designed to support literature reviews [25]. Retrieved references were added to the software for the purpose of duplicate checking as well as title and abstract screening. Duplicates identified by the software were removed. The identified studies were evaluated in full text for the purpose of final inclusion.

Data extraction was conducted on all the studies identified for full-text screening. To ensure consistency and comprehensiveness, a standardized data extraction template was created using Microsoft Excel. The data involved general information about each study, including authorship, year of publication, country of origin, study objectives, study design, study setting, and study results.

Aside from the overall study features, additional data elements were extracted to answer each of the 3 research questions. To answer the first research question on the types of AI tools, information was collected on the specific AI technologies applied in the studies (eg, neural networks, ML, and NLP).

For the second research question on the application of AI tools in infectious disease control, data were extracted regarding their use or purpose (eg, prediction, prevention, surveillance, and response to outbreak), the degree of implementation (eg, local, national, and regional), and the specific type of infectious disease being targeted.

Finally, to address the third research question on leadership and participation in AI-enabled public health initiatives, affiliation data of the authors were obtained, including the type of institution (eg, university, government, or company), academic or professional discipline (eg, medicine, nursing, technology, or informatics), and authorship (eg, first author or corresponding author), with particular attention to the inclusion of nursing professionals.

A critical appraisal of study quality was not considered according to the updated methodological JBI guidelines for scoping reviews [15].

### Ethical Considerations

Ethics approval was not sought, as this review did not involve human subjects in any form (no collection of primary data from human participants or no use of secondary data).

## Results

### Article Selection

**Figure 2.**
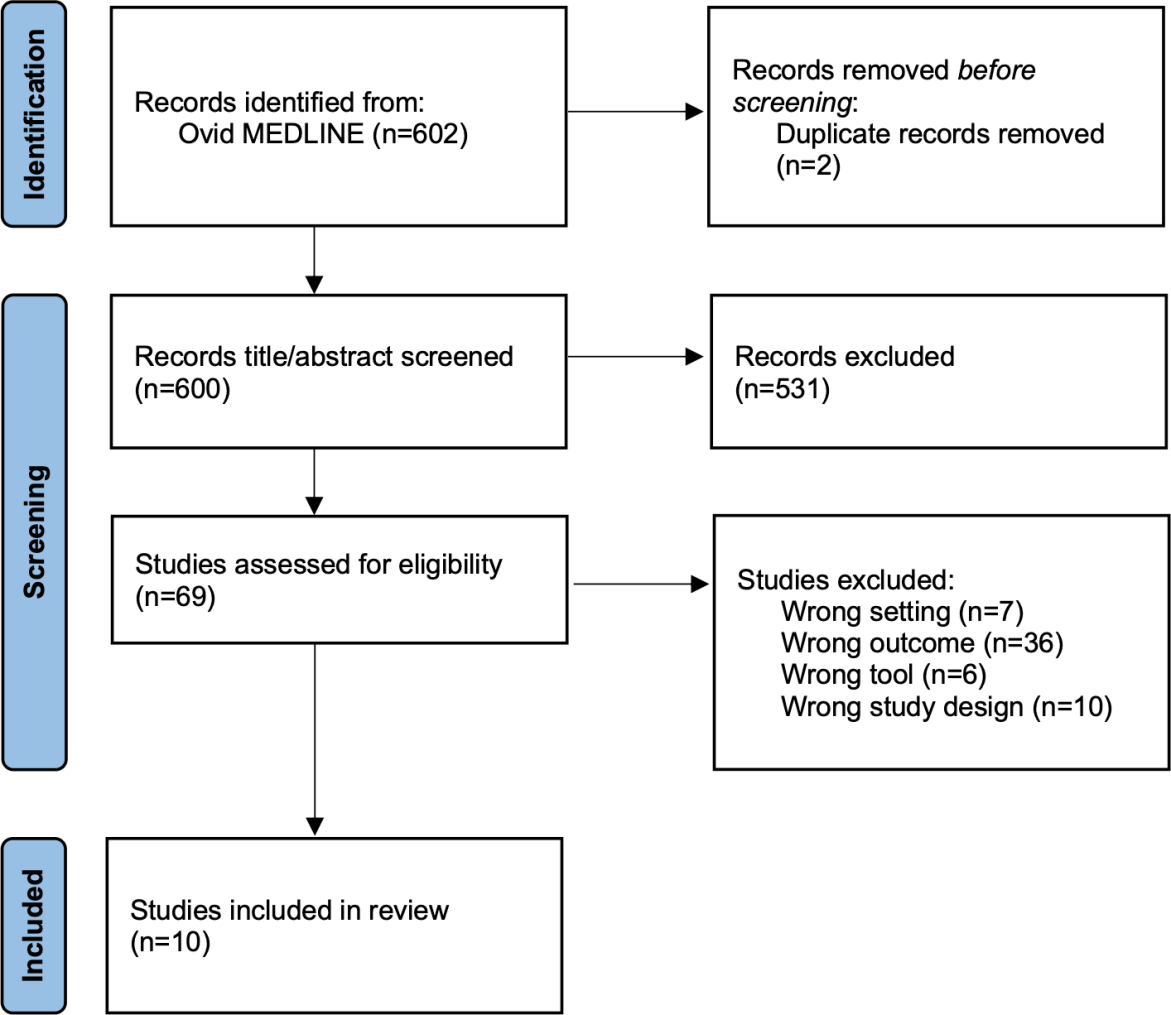
Article selection process.

The initial search in Ovid MEDLINE identified 602 records on June 13, 2025. After removing 2 duplicate records, 600 unique articles remained for screening. During title and abstract screening, 531 articles were eliminated primarily because they did not involve infectious diseases affecting humans. Of the remaining 69 studies, 59 were screened out at the full-text level for reasons such as nonpublic health focus (eg, clinic- or hospital-based studies), inappropriate study design (eg, protocols or editorials), irrelevant intervention (eg, tools not based on AI), or lack of real-world application (eg, studies limited to model development or simulation rather than implementation) (Figure 2). As a result, 10 studies were ultimately included in the final review. The PRISMA-ScR (Preferred Reporting Items for Systematic Reviews and Meta-Analyses extension for Scoping Reviews) checklist is presented in Checklist 1.

### General Characteristics of the Included Studies

Table 2 provides an overview of the key characteristics of the 10 articles that were selected for this review. These studies reflected a wide geographic distribution and covered several countries, including the United States (n=1), Brazil (n=2), Pakistan (n=1), Italy (n=2), China (n=1), Kenya (n=1), and Tanzania (n=1), with 1 study covering multiple countries in Africa (Burundi, Ethiopia, Madagascar, Uganda, Rwanda, and Zambia).

**Table 2.** Summary of key findings from the included studies.

Study	Country	Level of implementation	Target disease	Purpose of AI^a^
Asnake et al [26], 2025	Tanzania	National	VPDs^b^	Surveillance, vaccination
Cosma et al [27], 2025	Italy	Local	VPDs	Health communication
Friedman et al [28], 2025	Kenya	National	HIV	Screening
Gianquintieri et al [29], 2022	Italy	Local	COVID-19	Outbreak identification
Massa et al [30], 2023	Brazil	Local	HIV	Health education
Siddiqi et al [31], 2024	Pakistan	Local	VPDs	Vaccination, health education
de Souza Filho et al [32], 2023	Brazil	National	COVID-19	Screening
Tadese et al [33], 2024	Africa	Regional	VPDs	Surveillance, vaccination
Xiao et al [34], 2024	United States	Local	COVID-19	Resource allocation
Zhang et al [35], 2021	China	National	Foodborne diseases	Outbreak identification

a AI: artificial intelligence.

b VPDs: vaccine-preventable diseases.

In terms of scope, 5 studies used AI tools at the local level, targeting specific cities or subnational regions. Four studies were classified as national-level studies, including those that developed open-access web-based platforms without specifying a particular locality. The remaining study examined the regional usage of an AI tool and involved multiple African countries as mentioned above.

The target diseases of the included studies differed. Of the 10 studies, 3 addressed COVID-19, 4 addressed vaccine-preventable diseases, 2 focused on HIV, and 1 addressed foodborne diseases.

### Research Question 1: Types of AI Tools

The first research question examined what types of AI tools have been used to support the management of infectious diseases for public health. ML was the most utilized approach, and it appeared in 7 of the 10 studies. The remaining 3 studies utilized language-based AI technologies, including LLMs and NLP (Table 3).

**Table 3.** Summary of machine learning and language-based artificial intelligence studies.

Study	Software	Performance evaluation
Machine learning studies		
Asnake et al [26], 2025	Jupyter Notebook	Accuracy, precision, recall, *F*_1_-score, AUC^a^
Friedman et al [28], 2025	Likely implemented in R software	AUPRC^b^
Gianquintieri et al [29], 2022	Not specified	AUC
de Souza Filho et al [32], 2023	Not specified	Sensitivity, specificity, accuracy, AUC
Tadese et al [33], 2024	Jupyter Notebook	Accuracy, recall, *F*_1_-score, precision, AUC
Xiao et al [34], 2024	Likely implemented in R software	Reduction in hospitalization
Zhang et al [35], 2021	Python	Recall rate, *F*_1_-score
Language-based artificial intelligence studies		
Cosma et al [27], 2025	ChatGPT	—^c^
Massa et al [30], 2023	Dialogflow	—
Siddiqi et al [31], 2024	Python	—

a AUC: area under the curve.

b AUPRC: area under the precision-recall curve.

c Not applicable.

#### Language-Based AI: LLMs and NLP

Studies using language-based AI differed from those using ML in both applications of AI and data collection and analysis. These studies typically released chatbots or conversational platforms in real-world settings. One example is the study by Massa et al [30], which involved the development of “Amanda Selfie,” a gender-affirming chatbot that attempted to raise pre-exposure prophylaxis (PrEP) awareness among adolescents in Brazil. It was programmed to work on Facebook Messenger and used Dialogflow (Google LLC/Alphabet Inc), an NLP framework that allows the system to understand user messages as well as respond accordingly [36]. Dialogflow acts as the chatbot’s brain, determining user intent as well as managing conversation flow. Programming languages for the chatbot were Perl (Larry Wall/The Perl Foundation) and JavaScript (Brendan Eich/Oracle Corporation). Frameworks, such as Bottender, Catalyst, and Mojo, were used to ease and streamline the development process.

In another study, Siddiqi et al [31] developed “Bablibot,” a chatbot for caregivers of children who are eligible for immunization in Pakistan. Built with Python and the Django framework, it used NLP, ML, and a human-in-the-loop system combining AI automation with human oversight to provide real-time support in Roman Urdu via SMS text messages and WhatsApp messages. Bablibot was interoperable with the provincial immunization registry, providing customized responses, and could work without an internet connection, which made the project suitable for use in low-resource, low-literacy environments.

Another project used the readily available NLP-based AI model, ChatGPT, for generating and standardizing vaccination consent forms to be used in Italy [27]. In this study, the AI tool was used to provide customized information or recommendations depending on user needs. As a result, data for evaluating the effects of the AI tool were collected after deployment, based on actual user interactions.

#### ML Algorithms

Several studies employed ML to control infectious diseases from structured sets of data using supervised models of learning. As observed in studies by Tadese et al [33] and Asnake et al [26], model development began with data collection from large-scale, representative datasets, such as Demographic and Health Surveys (DHS), from which relevant features were extracted based on sociodemographic, maternal, and health care–related variables. In the study by Friedman et al [28], open-source geospatial datasets (eg, from WorldPop, Meta, or IHME) were also integrated to enrich individual-level medical data.

If needed, these datasets can be preprocessed through imputation of missing values; balancing techniques, such as SMOTE (Synthetic Minority Oversampling Technique); and normalization, so that ML models can learn properly scaled data [26,33]. However, if the ML model adopts the extreme gradient boosting (XGBoost) algorithm, which includes a built-in method called sparsity aware split finding, imputation is unnecessary because it enables handling missing values internally without prior imputation [28].

Building on this prepared data, model training is the follow-up step of tool development commonly mentioned in these studies, to enable accurate prediction or classification for a specified public health endpoint, for example, classifying children who are probable to have incomplete immunization or predicting zero-dose status [26,33]. In this step, various ML algorithms are commonly considered, for example, logistic regression, random forest (RF), support vector machine (SVM), XGBoost, and ensemble methodologies such as adaptive boosting. Tuning of hyperparameters is typically done as necessary for model performance optimization [26,28,33].

Common measures of evaluation included accuracy, precision, recall, and *F*_1_-score [33,26], as well as area under the curve (AUC) [35,32]. Additionally, Friedman et al [28] used area under the precision-recall curve, which is of particular significance under drastic class imbalance. The selection of measures normally conformed to the nature of the set and the intention of prediction. These measures were used to gauge the quality and strength of trained models for performing special public health tasks of prediction. They indicate how effectively the models identify, predict, or classify public health events in practice. For example, a higher AUC reflects stronger discriminatory ability to distinguish between at-risk and not-at-risk populations, while a higher *F*_1_-score suggests balanced model performance between sensitivity and precision, which is particularly valuable in public health decision-making.

Model interpretability is also a recurring focus. Tadese et al [33] and Zhang et al [35] used Shapley Additive Explanations (SHAP) values to quantify the input feature–specific contribution individually to model predictions, for use in supporting clinical or programmatic decision-making. In the study by Zhang et al [35], for instance, SHAP was used alongside tree-based models to identify salient risk factors for foodborne disease outbreaks from a national surveillance system across China. Additionally, Tadese et al [33] used SHAP for the interpretation of XGBoost predictors of incomplete immunization among children under the age of 5 years across East Africa. This assisted in distinguishing features that were common across underimmunized children. In each instance, the use of SHAP allowed for not only accurate but also interpretable prediction of modifiable risk factors and hence supported relevant public health responses.

Programming environments were stated explicitly, facilitating easier identification of development tools. Asnake et al [26] and Tadese et al [33] applied Jupyter Notebook, implying the use of Python. Zhang et al [35] also used Python, running their outbreak detection models with scikit-learn for model training and evaluation, XGBoost for boosting-based tree modeling, and fuzzywuzzy for string-based feature extraction.

In other cases, although the environment of programming itself was not mentioned, methodological information available suggests the likely selection of software. Xiao et al [34] likely ran their policy learning framework in R, as they closely followed the approach proposed by Athey and Wager, including the use of tree-based policy classes and doubly robust estimation [37]. Given that Athey and Wager provided R packages, such as policytree and grf, to support these procedures [37] and the model components of a similar nature (eg, finite-depth decision trees and generalized RFs) are also noted, the computational process of Xiao et al [34] strongly suggests an R-based implementation. Similarly, Friedman et al [28] did not indicate the use of software, but reference to the use of the mice package [38], an R package for handling multivariate imputation, suggests that R software was most likely used in their study.

### Research Question 2: AI Application Purposes in Infectious Disease Control

The type of AI (whether language- or ML-based) played an important role in the type of infectious disease control activities supported. This review found that studies that applied language-based models were primarily designed to support health communication and health education, while studies that involved ML-based models covered large-scale analysis of structured data tasks, such as surveillance, case identification, outbreak prediction, and resource mobilization, which have traditionally required more time and human labor (Table 2).

#### Health Communication and Health Education

Both Massa et al [30] and Siddiqi et al [31] described language-based AI technologies that were implemented to enhance health communication. The chatbots actively involved people, using individually tailored communication to prevent infectious diseases. Specifically, the Amanda Selfie chatbot provided more culturally appropriate messages for adolescent men who have sex with men and adolescent transgender women to promote awareness and use of HIV PrEP. Amanda Selfie adopted the language used by these adolescents. Interestingly, 61.1% of adolescents were reached through Amanda Selfie, higher than what would be achieved on Instagram or WhatsApp. This tool was especially effective among transgender adolescent girls and young people. Although its sole impact on the uptake of PrEP was modest, Amanda Selfie showed increased uptake when accompanied by professional support.

Similarly, Bablibot promoted vaccination by providing essential information for caregivers [31]. By addressing common vaccine schedule worries, adverse events, and service delivery, the tool helped reduce caregivers’ concerns regarding vaccination and promoted informed decisions. Its integration into the local immunization registry also supported customized communication based on children’s immunization history. This helped Bablibot reduce missed vaccinations and increase caregiver confidence regarding immunization programs, where traditional one-to-one counseling remained limited.

Cosma et al [27] used ChatGPT for improving the usability and readability of vaccination consent forms as an alternative health communication. ChatGPT revised medical technical terms and abbreviated formal forms for ease of reading for patients and family members. These forms were subsequently evaluated by health care professionals in the vaccination unit, showing improved usability and readability. The AI tool served as an assistant for reducing the workload of manually preparing user-friendly health papers.

#### Identifying At-Risk Individuals or Populations

One of the significant strengths of ML is its ability to process large-scale data and extract complex patterns that would otherwise be unavailable using traditional statistical methods [39]. This makes ML an ideal candidate for identifying individuals or subgroups most vulnerable and those needing special consideration. For instance, Asnake et al [26] and Tadese et al [33] used supervised learning models, including XGBoost and RF, to analyze DHS data and predict incomplete or zero-dose vaccination status among children in East Africa. By identifying risk profiles, including maternal education, household income, antenatal care visits, and geographic location, the models presented actionable suggestions about subgroups that require the most attention for immunization outreach.

Friedman et al [28] demonstrated a similar approach for the scenario of HIV screening in Kenya. Based on routinely and officially collected electronic medical record data, they trained multiple supervised learning algorithms to identify individuals likely to test positive for HIV. Their model had remarkable predictive accuracy and was constructed for implementation into real-time clinical practice, with high suitability for resource-challenged settings. Hence, the system makes the most of the efficiency of the tests, optimizes service prioritization, and minimizes missed diagnoses.

One of the studies adopted probable case identification of COVID-19 using a digital screening platform [32]. The online platform used an ML algorithm to estimate the likelihood of COVID-19 based on user-reported symptoms and sociodemographic characteristics. Integrated into a telemedicine service, the platform helped reduce unnecessary hospital referrals by classifying patients requiring further evaluation. Deployed in Brazil during the early stages of the pandemic, it served as both a triage support system and an early warning mechanism for local outbreak control.

#### Early Warning of Disease Outbreaks

ML’s capacity to process large and complex data is useful in various types of disease surveillance, including early warning of disease outbreaks. Zhang et al [35] developed a high-performance algorithm to overcome the large number of false positives of suspected outbreaks reported by China’s national Foodborne Disease Monitoring and Reporting System. Among various ML algorithms that they explored, the XGBoost model achieved the highest performance, showing an excellent capability of distinguishing between true outbreaks and false alarms. Notably, the model output included not only binary classifications but also probability scores, enabling fine-grained alert levels.

Meanwhile, Gianquintieri et al [29] applied ML for emergency medical service records to create high-resolution territorial notifications for COVID-19 across Lombardy, Italy. The supervised learning algorithm (RF) reached approximately 80% sensitivity for identifying active transmission and provided daily, municipality-scale notifications capable of preceding official outbreak patterns. This approach facilitated more proactive public health responses and effective prioritization of testing and health care resources, particularly for initial or resurging pandemic waves.

#### Resource Allocation

Xiao et al [34] showed that the ML algorithm is also applicable for optimized resource allocation in a public health crisis. They developed a policy learning tree (PLT) model using retrospective electronic health record data to improve the distribution of monoclonal antibodies for COVID-19 treatment in the United States. The PLT model was trained to focus treatment on patients who would most benefit from treatment and minimize hospitalization. By comparison, the ML-based approach had higher accuracy and efficiency than traditional point-based strategies, and it has the potential to inform real-time clinical decision-making for public health emergencies.

### Research Question 3: Leadership and Participation in AI-Enabled Public Health Efforts

To examine who is leading and participating in AI-enabled public health interventions, the institutional affiliations, disciplinary backgrounds, and authorships of the 10 selected studies were analyzed.

A total of 77 authors contributed to the 10 studies included in this review. Among them, 3 individuals held dual affiliations, resulting in 80 total institutional affiliations. As shown in Table 4, academic institutions accounted for the largest share (47/80, 59%), followed by government agencies (14/80, 18%), private companies (7/80, 9%), nongovernmental organizations (NGOs) (7/80, 9%), clinics (3/80, 4%), and independent research institutes (2/80, 3%).

**Table 4.** Institutional affiliations of authors contributing to artificial intelligence–enabled public health interventions.

Type of institution	Value (N=80^a^), n (%)
University	47 (59)
Government	14 (18)
Company	7 (9)
NGO^b^	7 (9)
Clinic	3 (4)
Research institute	2 (3)

a Three authors had dual affiliations; therefore, the total number of institutional affiliations (N=80) exceeds the total number of authors (N=77).

b NGO: nongovernmental organization.

The disciplinary classification of each author’s affiliation was then analyzed. Of the 78 disciplinary designations identified among the 77 authors (accounting for dual affiliations), the most common area was public health, accounting for 34 out of 78 cases (44%). This was followed by informatics (17 affiliations, 22%) and medicine (15 affiliations, 19%). Technology-related disciplines accounted for only 5 affiliations (6%), while 7 affiliations (9%) fell into the “other” category (Table 5).

**Table 5.** Disciplinary classification of authors’ affiliations.

Disciplinary field	Value (N=78^a^), n (%)
Public health	34 (44)
Informatics	17 (22)
Medicine	15 (19)
Technology	5 (6)
Other	7 (9)

a Dual affiliations in different disciplinary fields resulted in a total of 78 disciplinary designations, although there were 77 authors in total.

**Figure 3.**
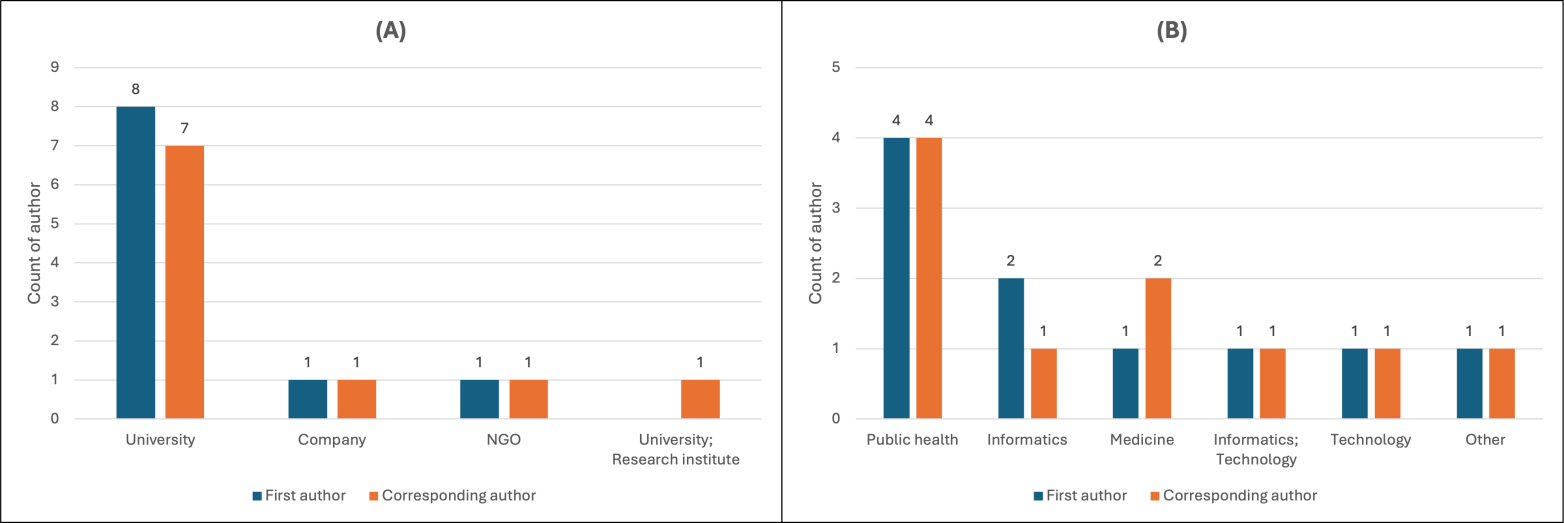
Author role distribution by institution type (A) and discipline (B) in artificial intelligence–enabled infectious disease control studies. NGO: nongovernmental organization.

Authorship roles were also explored to examine how leadership in AI-enabled public health interventions is distributed. Universities again comprised most first and corresponding author affiliations. Among the 10 studies, 8 had first authors from universities and 7 had corresponding authors from universities, followed by NGOs and an independent research institute (Figure 3A). Disciplinary affiliations also showed that public health was the most frequently represented field across both roles (Figure 3B). Informatics-related departments were more frequent for first authors, whereas medical departments were more likely for corresponding authors. There were no nursing-affiliated authors as first authors or corresponding authors, and the analysis of all co-authors did not indicate their presence.

## Discussion

### Principal Findings

This review aimed to map the use of AI for infectious disease control within public health settings in the post–COVID-19 era. It focused on identifying cases of actual implementation in real-world contexts. By synthesizing evidence from 10 studies across diverse global settings, it highlighted the types of AI tools used, their applications in infectious disease control, and the profiles of the experts leading these efforts.

#### AI for Infectious Disease Control: Language Models Versus ML

One valuable finding was the clear division of AI applications into 2 major categories: ML-based modeling and language-based communication tools. This distinction reflects broader structural dynamics in public health, where back-end analytics for decision-making and front-line community engagement for health communication often function as separate operational domains. ML tools like RF, SVM, and XGBoost, were frequently applied to structured datasets to perform classification, prediction, and risk stratification. These approaches facilitated the timely detection of populations at increased risk [26,28,33], outbreak forecasting [29,35], and data-driven resource allocation [34].

By contrast, language-based systems, such as chatbots and LLMs, were used primarily to enhance communication, education, and user engagement. These tools often operated on popular platforms like Facebook Messenger or WhatsApp, allowing real-time interaction in local languages and formats tailored to specific populations [30,31]. Their deployment was evident in interventions focused on vaccine promotion or HIV prevention, where timely and culturally responsive communication is essential [30,31].

Notably, these 2 categories vary in terms of not only function but also technological accessibility. AI implementation typically demands considerable technological infrastructure and expertise, including programming expertise, statistical modeling familiarity, and the ability to work with large-scale health data [40]. This might in part account for the fact that these tools were largely created within research institutes or universities. Conversely, language-based tools lend themselves more easily to implementation using available chatbot frameworks, application programming interface–based services, and commercial applications like ChatGPT. These systems usually demand no or little programming expertise and are thus more accessible for public health practitioners with limited technological capability [41]. As such, the lower barrier to entry for language-based AI might have made it easier to broadly adopt these technologies in real-world public health contexts.

However, the studies included in this review largely concerned text- and data-based modalities. While different forms of multimodal AI, including visual, behavioral, and biometric instruments, are becoming increasingly available [42], no multimodal AI studies were identified that involved application in practice within real-world public health, implying a limited technological scope within practice at present.

Some experimental research was found during the initial screening, which indicates potential directions. For instance, a study created an AI system to track people’s social distancing in low-light situations and presented a possible solution for pandemic real-time behavioral tracking [43]. Other innovations included automated drone-based mosquito release systems [44] and mobile instruments for determining the species of mosquitoes based on sound recordings [45]. Although these studies were excluded from the final analysis due to limited real-world implementation, these examples show upcoming prospects for expanding AI use beyond text and data.

#### Two Functions of AI in Infectious Disease Control: Communication and Analysis

Another important finding of this review was that the functional applications of AI in the prevention and control of infectious diseases broadly fall into two main categories: (1) health communication and education, and (2) data-driven analysis and decision support. These functions were closely aligned with the type of AI technology employed.

Health communication and education emerged as prominent areas where AI was applied. Language-based tools, such as chatbots and LLMs, were commonly used to facilitate real-time messaging, disseminate accurate information, and encourage behavior change. These tools often operated on popular platforms like Facebook Messenger or WhatsApp [30,31]. Cosma et al [27] further demonstrated that tools, like ChatGPT, can be not only effective but also easily implemented even by users with minimal technical expertise.

Meanwhile, ML was used to process large-scale datasets and inform decision-making and strategic planning. ML models were frequently used to analyze data from national surveillance systems, medical records, and health surveys. These models supported outbreak detection [29,35], identification of at-risk populations [26,28,32,33], and allocation of resources [34].

This functional divide suggests that the roles of AI in public health are shaped by not only technical capabilities but also the specific demands of the intervention context. Language-based AI excels in fostering user engagement and information exchange, whereas ML algorithms are better suited for handling complexity and scale in backend decision-making. While the former typically interfaces with the public, the latter primarily supports health professionals behind the scenes.

These findings are well aligned with the WHO’s 5Cs framework, which was initially used to delineate the scope of public health interventions in this review. Language-based AI primarily supports community protection through communication and education, while ML contributes to collaborative surveillance, emergency coordination, and access to countermeasures by enabling data-driven prediction, response, and resource management across public health systems. Although safe and scalable care was not directly addressed in the reviewed studies, it remains an important domain for future AI applications in clinical and workforce settings.

#### Leadership Without Nurses? Participation Patterns in AI-Based Interventions

The authorship analysis revealed clear patterns regarding who is leading and participating in AI-enabled public health interventions. Academic institutions dominated authorship roles, accounting for the majority of both first and corresponding authors. Disciplines, such as public health, informatics, and medicine, were most represented, with technical and analytical expertise concentrated among first authors, and medical leadership more often reflected among corresponding authors.

However, one of the most striking findings was the complete absence of nursing professionals in primary authorship positions or among the broader group of co-authors affiliated with schools, colleges, or departments of nursing across the 10 final studies. This is surprising given the central role nurses play in health systems globally, particularly in frontline service delivery, community health engagement, and population-level care coordination [14].

Importantly, this absence does not appear to be limited to public health alone. A broader body of literature indicates that nurses are frequently absent from the design and decision-making structures for digital health technologies in other areas, such as clinical decision support, predictive analytics, and patient-facing AI [46,47]. As Bakken and Dreisbach argue [48], the incorporation of nursing insights is essential for creating more holistic health information systems.

As noted earlier, nurses comprise approximately 50% of the global health workforce, and there are an estimated 29 million nurses worldwide [14]. The limited involvement of nurses in the development and implementation of AI and other digital health technologies is an important issue that needs to be addressed. This underrepresentation may be partly explained by structural challenges like the unavailability of AI education or data infrastructure in nursing curricula or by further isolation of nursing expertise from technological innovation areas [48]. The American Association of Colleges of Nursing and the Alliance for Nursing Informatics are advocating for informatics/AI competencies in nursing education and advancing nursing informatics leadership, including AI projects, but gaps remain due to the need for training, curriculum revisions, and limited funding opportunities. The current gaps highlight an urgent need for more intentional inclusion of nursing voices within interprofessional teams developing and using AI-based technologies for public health and health science.

### Recommendations to Address Gaps in Implementation and Representation

#### Gaps

Despite encouraging innovations, this review highlights several notable gaps. First, this review identified only 10 studies that implemented AI tools in real-world public health practice—a number vastly lower than anticipated, considering the number of AI research articles that get published at present. Second, the applications of AI were not evenly spread across regions and disease conditions. The majority were concerned with COVID-19 or vaccine-preventable infections, while other infectious diseases like tuberculosis and malaria still carry a large burden across the world, primarily in low- and middle-income countries [49,50]. Further, scaling up at national or international levels was less frequent, and this indicates low scalability for most tools beyond pilot scaling at local levels.

#### Strengthen Implementation Research on AI in Public Health Practice

The relative absence of implementation studies in public health indicates that most AI innovations exist at the pilot or proof-of-concept phase, with few attaining system-level implementation. To get a complete view of the feasibility, sustainability, and public health effects of AI-enabled interventions, more implementation research is needed. This involves exploring how AI tools get implemented, adapted, and scaled within the framework of the existing health system. Strengthening this body of work is essential to bridge the gap between AI-based tool development and the public health improvement desired, and to ensure that technological innovations translate into measurable improvements in disease prevention and control.

Next, and most importantly, there was no representation of nursing professionals as authors within the analyzed literature. Considering the important contributions of nurses to the health system, this omission is an important oversight. This oversight could affect the practicality and fairness of AI-enabled interventions in the real world. The complete absence of professionals affiliated with nursing programs or nursing-focused organizations in authorship roles, despite nurses’ central roles in frontline health protection, is a significant finding for additional action.

#### Advance Informatics Education to Support Nursing Participation in AI

While educational background could not be assessed directly, this absence may underscore the need for formal informatics education and AI training in nursing, which includes foundational data literacy, ethics of AI, and basic principles of human-AI interaction. Future research must examine what the most ideal type of educational intervention, curricular revision, or continuing education is for equipping nurses with the baseline skills needed for contribution back into AI development and application. Empowering nurses with the ability to meaningfully interact with AI is not only a matter of workforce development but also a strategic imperative for ensuring that emerging technologies are grounded in the realities of care delivery and aligned with the values of person-centered, equitable public health.

### Limitations

This review has several limitations. First, the search was limited to a single database (Ovid MEDLINE) and excluded studies indexed in other bibliographic databases (eg, Scopus, Web of Science, and IEEE Xplore). Hence, the results and subsequent interpretations are based on studies indexed in MEDLINE, which limits the comprehensiveness of the review, likely missing relevant studies in computer science or broader multidisciplinary databases. Although this strategy assisted in increasing the specificity of the search, it decreased the generalizability of the findings.

Second, the inclusion criterion was the use of AI tools in practical public health situations. Therefore, many simulation- or model-based studies were excluded. However, they could be helpful for future implementation in epidemic forecasting, as explained by Kraemer et al [51]. While the eligibility criterion in this study helped prioritize studies with demonstrated practical application, it may have narrowed the scope of technologies captured in the review.

Third, the disciplinary categorization of authors aligned with institutional affiliation and not with individual professional education. Therefore, the lack of nursing professionals could be an indication of the lack of metadata available and not the absolute absence of nursing engagement. In this way, the results for professional representation must be interpreted with care because the results could be affected by how disciplinary affiliation is reported and documented.

### Conclusion

AI technologies are increasingly being applied to support public health responses to infectious diseases, with applications ranging from predictive analytics to real-time public engagement. However, adoption remains limited in scale, scope, and professional diversity. The near-total absence of nursing participation in AI-related public health research is particularly striking and represents a missed opportunity for inclusive innovation. Strengthening implementation research and advancing informatics education among nursing professionals are critical next steps to ensure that AI tools are equitable, reflect the realities of public health practice, and promote equitable outcomes.

## Supplementary material

10.2196/84242 Multimedia Appendix 1 Search strategy.

10.2196/84242 Checklist 1 PRISMA-ScR checklist.

## Abbreviations

AI: artificial intelligence
AUC: area under the curve
DHS: Demographic and Health Surveys
JBI: Joanna Briggs Institute
LLM: large language model
MeSH: Medical Subject Headings
ML: machine learning
NGO: nongovernmental organization
NLP: natural language processing
PCC: population, concept, and context
PLT: policy learning tree
PrEP: pre-exposure prophylaxis
RF: random forest
SHAP: Shapley Additive Explanations
SVM: support vector machine
WHO: World Health Organization
XGBoost: extreme gradient boosting
