# Supplementary material for: Applications of Artificial Intelligence in the Control of Infectious Diseases in the Post-COVID Era: Scoping Review
Source: JMIR Nurs. 2025 Nov 17;8:e84242. doi: 10.2196/84242 (PMC12622858; doi:10.2196/84242)
Supplement: Multimedia Appendix 1 [file nursing-v8-e84242-s001.docx]

**Ovid MEDLINE(R) ALL <1946 to June 13, 2025>**

| No | Searches | Results |
| --- | --- | --- |
| 1 | exp *Artificial Intelligence/ or (artificial intelligence or AI or large language model* or machine learning or deep learning or natural language processing or neural network* or generative artificial intelligence or genAI or ChatGPT* or chatbot*).ti. | 250401 |
| 2 | exp *Communicable Disease Control/ or exp *Immunization Programs/ | 255299 |
| 3 | (exp *Disease Transmission, Infectious/ or exp *communicable diseases/ or exp *Disease Outbreaks/ or exp *COVID-19/) and (exp *Disaster Planning/ or exp *Public Health Practice/ or exp *Health Services/ or exp *Health Communication/ or exp *Public Health Administration/ or exp *"Delivery of Health Care"/ or exp *Diagnostic Tests, Routine/ or exp *Early Diagnosis/ or exp *Pharmaceutical Preparations/ or exp *Drug Delivery Systems/ or exp *Resource Allocation/) | 130451 |
| 4 | 2 or 3 | 353734 |
| 5 | 1 and 4 | 1114 |
| 6 | limit 5 to (english language and yr="2020 -Current" and "humans only (removes records about animals)") | 722 |
| 7 | limit 6 to (case reports or comment or editorial or letter or meta analysis or news or "review" or "scoping review" or "systematic review") | 120 |
| 8 | 8 6 not 7 | 602 |
